# CGK733-induced LC3 II formation is positively associated with the expression of cyclin-dependent kinase inhibitor p21^Waf1/Cip1^ through modulation of the AMPK and PERK/CHOP signaling pathways

**DOI:** 10.18632/oncotarget.5625

**Published:** 2015-10-13

**Authors:** Yufeng Wang, Yasuhiro Kuramitsu, Byron Baron, Takao Kitagawa, Kazuhiro Tokuda, Junko Akada, Kazuyuki Nakamura

**Affiliations:** ^1^ Department of Biochemistry and Functional Proteomics, Yamaguchi University Graduate school of Medicine, Ube, Japan; ^2^ Centre for Molecular Medicine and Biobanking, Faculty of Medicine and Surgery, University of Malta, Msida MSD2080, Malta; ^3^ Centre of Clinical Laboratories in Tokuyama Medical Association Hospital, Shunan, Japan

**Keywords:** CGK733, LC3, p21, PERK/CHOP, AMPK

## Abstract

Microtubule-associated protein 1A/1B-light chain 3 (LC3)-II is essential for autophagosome formation and is widely used to monitor autophagic activity. We show that CGK733 induces LC3 II and LC3-puncta accumulation, which are not involved in the activation of autophagy. The treatment of CGK733 did not alter the autophagic flux and was unrelated to p62 degradation. Treatment with CGK733 activated the AMP-activated protein kinase (AMPK) and protein kinase RNA-like endoplasmic reticulum kinase/CCAAT-enhancer-binding protein homologous protein (PERK/CHOP) pathways and elevated the expression of p21^Waf1/Cip1^. Inhibition of both AMPK and PERK/CHOP pathways by siRNA or chemical inhibitor could block CGK733-induced p21^Waf1/Cip1^ expression as well as caspase-3 cleavage. Knockdown of LC3 B (but not LC3 A) abolished CGK733-triggered LC3 II accumulation and consequently diminished AMPK and PERK/CHOP activity as well as p21^Waf1/Cip1^ expression. Our results demonstrate that CGK733-triggered LC3 II formation is an initial event upstream of the AMPK and PERK/CHOP pathways, both of which control p21^Waf1/Cip1^ expression.

## INTRODUCTION

Microtubule-associated protein 1A/1B-light chain 3 (LC3)/autophagy-related gene 8 (ATG8), which has three isoforms in humans (LC3A, LC3B, and LC3C), is a ubiquitin-like protein required for autophagosome formation [1–4]. Lysosomal turnover of LC3-II can directly mirror autophagic activity, and detecting LC3 by immunoblotting or immunofluorescence is so far a reliable method for monitoring autophagy and autophagy-related processes [5]. Recently, LC3 has also been found to be critical for other pathophysiological vesicular pathways [6–8].

LC3 directly binds with protein 62 (p62/SQSTM1) resulting in the autophagy-independent protein aggregation and LC3-puncta formation [9, 10]. However, some studies have shown that immunoblotting of LC3 II may be helpful to resolve the problem of discerning what is and what is not autophagy [10, 11].

CGK733 has been reported to be involved in cell growth in drug-induced senescent tumor cells and trigger apoptotic death [12]. In our present study, we investigate the effects of CGK733 on the intracellular signaling pathways and autophagy. We show that CGK733 induced LC3 II and LC3-puncta formation in an autophagy-independent manner. Following treated with CGK733, there is no alteration in the autophagic flux and p62-mediated degradation. Interestingly, however, the accumulated LC3 II directly triggers the activation of AMP-activated protein kinase (AMPK) and the protein kinase RNA-like endoplasmic reticulum kinase/CCAAT-enhancer-binding protein homologous protein (PERK/CHOP) signaling pathway, as well as expression of p21^Waf1/Cip1^.

## RESULTS

### CGK733 induces LC3 II and LC3-puncta formation

**Figure 1:**
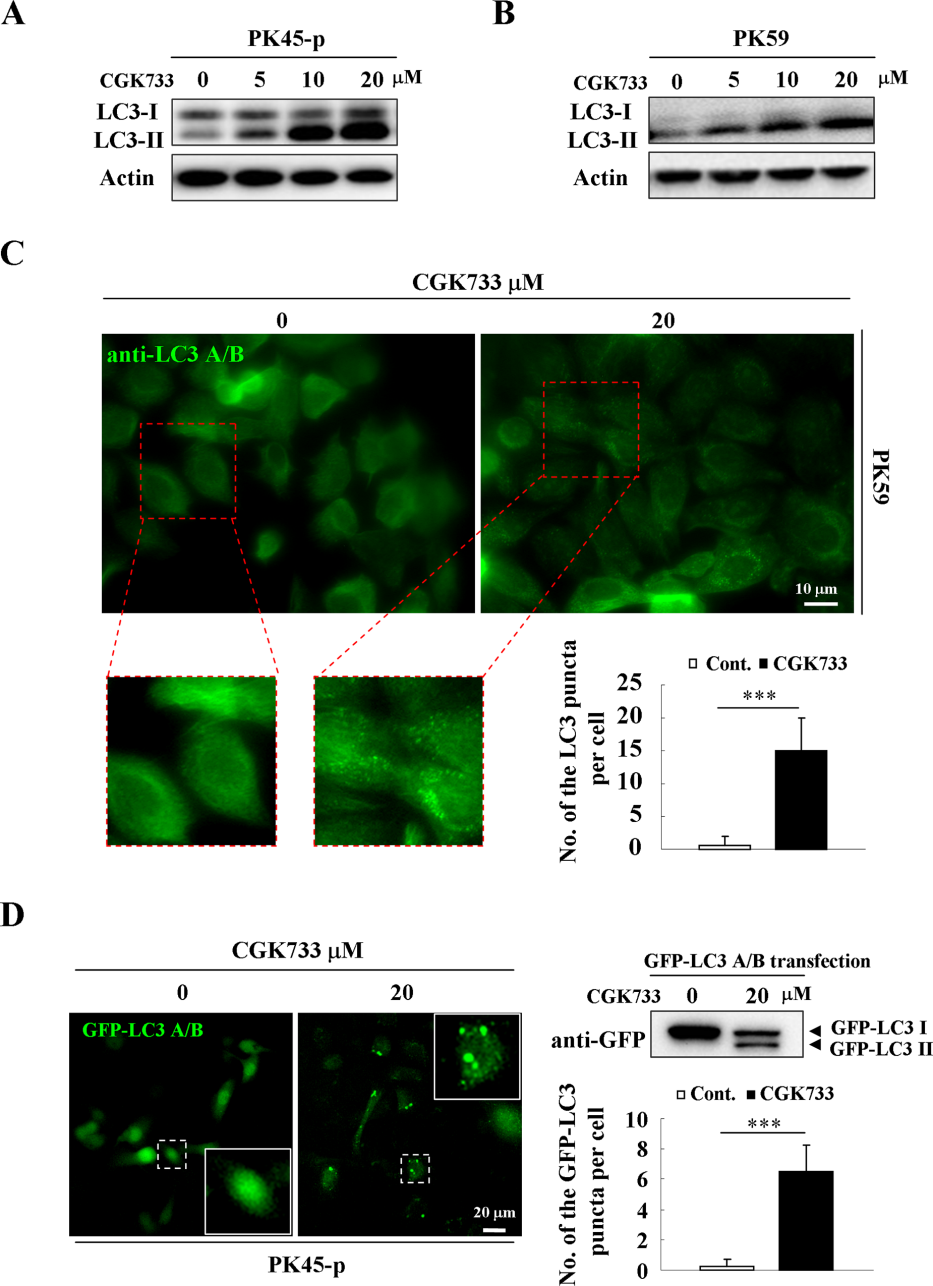
CGK733 induces LC3 II formation in Gemcitabine-resistant pancreatic cancer cells **A.** PK45-p and **B.** PK59 cells were treated with CGK733 for 6 h before being processed for Western blot analysis using antibodies against LC3 A/B and actin. **C.** PK59 cells were treated with CGK733 for 6 h before being processed for immunofluorescence using an antibody against LC3 A/B. Quantification of the samples are expressed as the number of endogenous LC3 puncta per cell. **D.** PK45-p cells were transfected with TagGFP2-LC3 Lentivirus for 48 h and then treated with CGK733 for 6 h before observation by fluorescent microscopy. Quantification of the samples are expressed as the number of GFP-LC3 puncta per cell. Error bars represent the standard deviations from counting 30 cells in three independent experiments. ***, *p* < 0.001.

To test if CGK733 induces LC3 II and LC3-puncta formation, immunoblotting and immunofluorescent assays were performed using anti-LC3 antibodies, following GFP-LC3 gene transfection. A significant up-regulation of the LC3-II bands resulted from treatment of pancreatic cancer cell lines with CGK733 for 6 h, in a dose-dependent manner (Figure 1A and 1B). The LC3-puncta assay was performed by using two different methods (GFP-LC3 transfection and anti-LC3 antibody staining) in PK45-p and PK59 cells respectively. The number of LC3-puncta was shown to be significantly increased after cells were exposed to CGK733 for 6 h compared to the non-treated controls (Figure 1C and 1D). This result was also obtained in healthy NIH 3T3 cells after treatment with CGK733 for 6 h (Supplementary Figure S1). LC3-II is the lipidated form of LC3 that specifically correlates with autophagosome membranes, and has been widely used to study autophagy as an autophagosome marker [1, 13]. In order to confirm the role of LC3 in our experiment, we next queried whether CGK733 induces autophagy.

### CGK733-induced LC3 II formatiom is autophagy-independent

**Figure 2:**
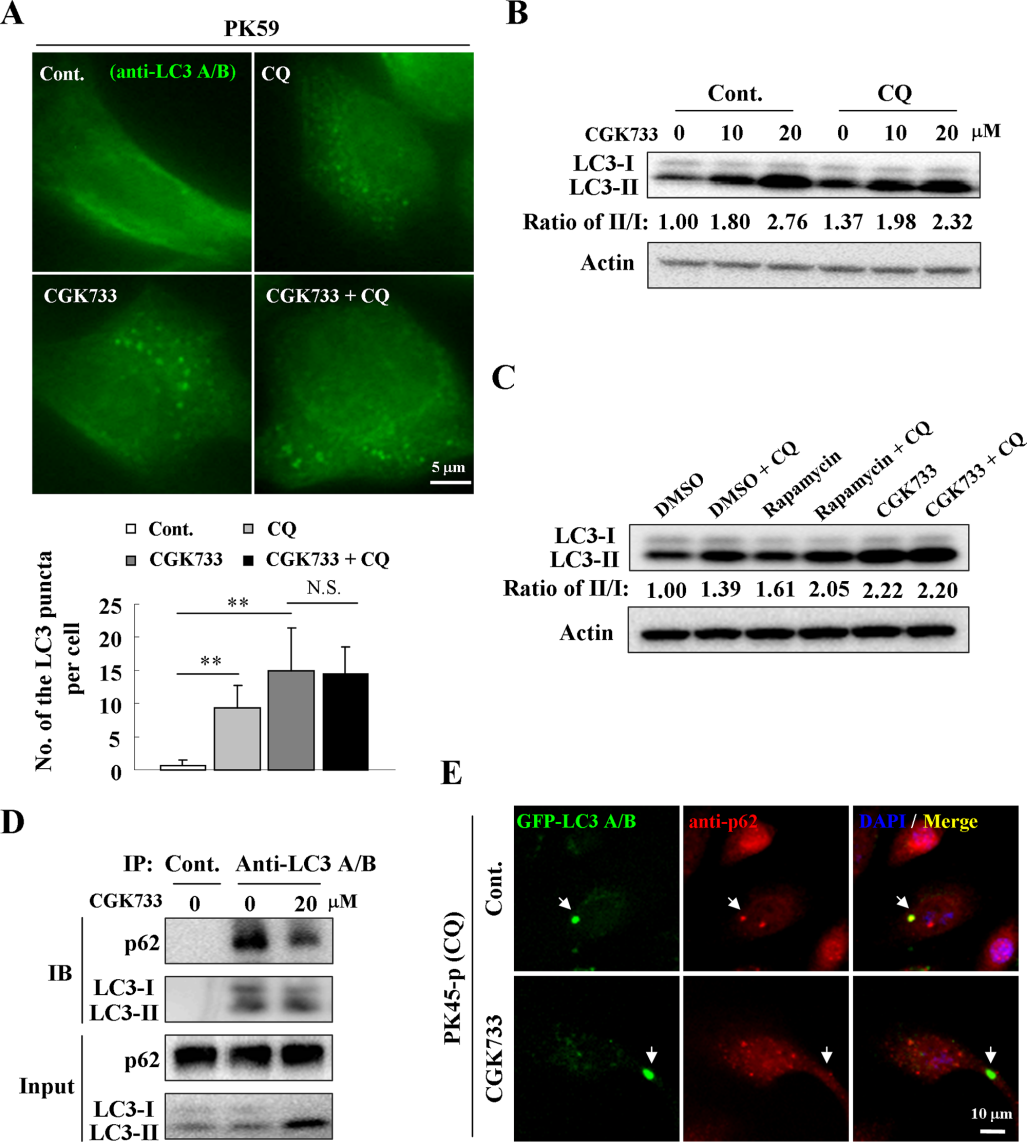
CGK733 failed to increase the autophagic flux and p62-mediated degradation **A.** PK59 cells were treated with CGK733 for 6 h in the presence or absence of 10 μM of CQ before analysis by immunofluorescence using an antibody against LC3 A/B. The lower figure indicates the related quantification of the endogenous LC3 puncta per cell. Error bars represent the standard deviations from counting 30 cells in three independent experiments. **, *p* < 0.01. **B.** PK45-*p* cells were treated with CGK733 for 6 h in the presence or absence of 10 μM of CQ before analysis by Western blot. **C.** PK59 cells were treated with CGK733 or rapamycin for 6 h in the presence or absence of 10 μM of CQ before analysis by Western blot. **D.** PK45-*p* cells were treated with CGK733 for 6 h before performing immunoprecipitation using an antibody against LC3 A/B. 1% of the original samples were used as inputs. **E.** PK45-*p* cells were transfected with TagGFP2-LC3 Lentivirus for 48 h and then treated with CGK733 before analysis by immunofluorescence using an antibody against p62. CQ was added in this experiment in order to easily observe autophagosomes in non-treated cells.

Since autophagy is an intracellular degradation system that transports cytoplasmic materials to the lysosomes [14], we tested whether GCK733 can activate the autophagic flux and stimulate autophagic degradation. Chloroquine (CQ), which causes lysosome dysfunction and blocks fusion of the autophagosome with the lysosome [15], was used to detect autophagic flux during treatment with CGK733. Autophagosomes accumulated in PK59 cells following CQ treatment compared to the untreated control (Figure 2A). However, no significant difference was observed in the number of LC3-puncta formed in cells after treatment with CGK733 (20 μM) in combination with CQ compared to CGK733 alone by an LC3-puncta assay (Figure 2A). Immunoblotting was also performed to validate the accumulation of LC3-lipidation by treatment with CGK733 in combination with or without CQ. Expectedly, CQ could not enhance the LC3-lipidation induced by treatment with 20 μM of CGK733 (Figure 2B and 2C) (although it could with just 10 μM of CGK733). It is possible that 10 μM of CGK733 might not be enough to overcome the intrinsic autophagy by CQ causing the enhancement of LC3-lipidation. These results indicate that CGK733-induced LC3-puncta structures may lack a mechanism for auto-lysosomal formation (fusion and degradation by lysosomes). On the other hand, the p62 protein (SQSTM1) is a ubiquitin-binding scaffold protein that holds ubiquitinated proteins to the autophagosomes by binding directly to LC3 during autophagosome-lysosome degradation [16]. Therefore we analyzed the levels of p62 expression as well as total intracellular ubiquitination and p62 binding with LC3 after cells were exposed to CGK733. The expression of p62 was not reduced and total protein ubiquitination was not altered by CGK733 (Supplementary Figure S2). Co-immunoprecipitation showed that p62-LC3 binding was not elevated (but diminished) after cells were exposed to CGK733 for 6 h (Figure 2D). Likewise, the co-localization of p62 and GFP-LC3 dots could not be observed following CGK733 treatment, but was present in the untreated control (Figure 2E). CQ was used in this experiment to visualize LC3-puncta in untreated cells. These data indicate that CGK733-induced LC3 II and LC3-puncta formation were not accompanied by p62 co-operation. Taken together, these results demonstrate that the CGK733-induced autophagy-like event does not have the essential features or function of canonical autophagy.

### CGK733 up-regulates P21^Waf1/Cip1^ through AMPK and the PERK/CHOP signaling pathway

**Figure 3:**
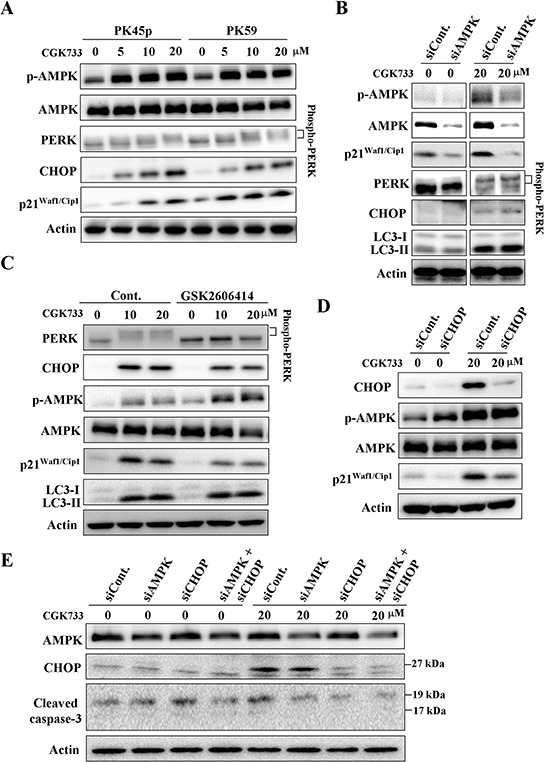
CGK733 induced AMPK phosphorylation and PERK/CHOP activation **A.** PK45-*p* and PK59 cells were treated with CGK733 for 6 h before being processed for Western blot analysis. **B.** PK45-*p* cells were treated with CGK733 for 6 h after transfection with AMPK siRNA for 48 h. **C.** PK45-*p* cells were treated with CGK733 for 6 h in the presence or absence of 1 μM of PERK inhibitor (GSK2606414) before analysis by Western blot. **D.** PK45-*p* cells were treated with CGK733 for 6 h after transfection with CHOP siRNA for 48 h. **E.** PK45-*p* cells were treated with CGK733 for 6 h after transfection with AMPK and/or CHOP siRNAs for 48 h.

Interestingly, treatment with CGK733 greatly activated AMPK (phospho-AMPK at Thr 172), the PERK/CHOP signaling pathway and the expression of p21^1/Cip1^ (Figure 3A). AMPK phosphorylation (at Thr 172), is essential for the activation of AMPK, and is required for autophagy by inhibiting mammalian target of Rapamycin (mTOR)-dependent signaling [17]. PERK facilitates survival of extracellular matrix-detached cells by concomitantly promoting autophagy [18]. We then tested whether AMPK phosphorylation and/or PERK mediated LC3 II formation were induced by the treatment with CGK733. Cells were transfected with AMPK siRNA or treated with PERK inhibitor (GSK2606414) before being exposed to CGK733. Immunoblotting showed that neither knockdown of AMPK nor inhibition of PERK altered the CGK733-induced LC3 II formation (Figure 3B and 3C). However, both of them blocked the CGK733-induced expression of p21^Waf1/Cip1^ (Figure 3B and 3C). We further showed that knockdown of PERK`s downstream executor, CHOP, by siRNA also failed to induce LC3 II formation, but blocked the expression of p21^Waf1/Cip1^ induced by the treatment with CGK733 (Figure 3D). Moreover, inhibition of the PERK/CHOP signaling pathway by PERK inhibitor or CHOP siRNA could increase as well as enhance the CGK733-inducecd AMPK phosphorylation (Figure 3C and 3D), indicating a negative role for PERK/CHOP in AMPK activation. In addition, CGK733-induced cleavage of caspase-3 was diminished when both AMPK and CHOP were inhibited by siRNAs (Figure 3E). Thus, LC3 II formation is not consequently downstream of CGK733-induced AMPK and PERK/CHOP activation. These results indicate that CGK733 induces the expression of p21^Waf1/Cip1^ through the AMPK and PERK/CHOP signaling pathways.

### LC3 B is required for the CGK733-induced activation of AMPK and PERK/CHOP

**Figure 4:**
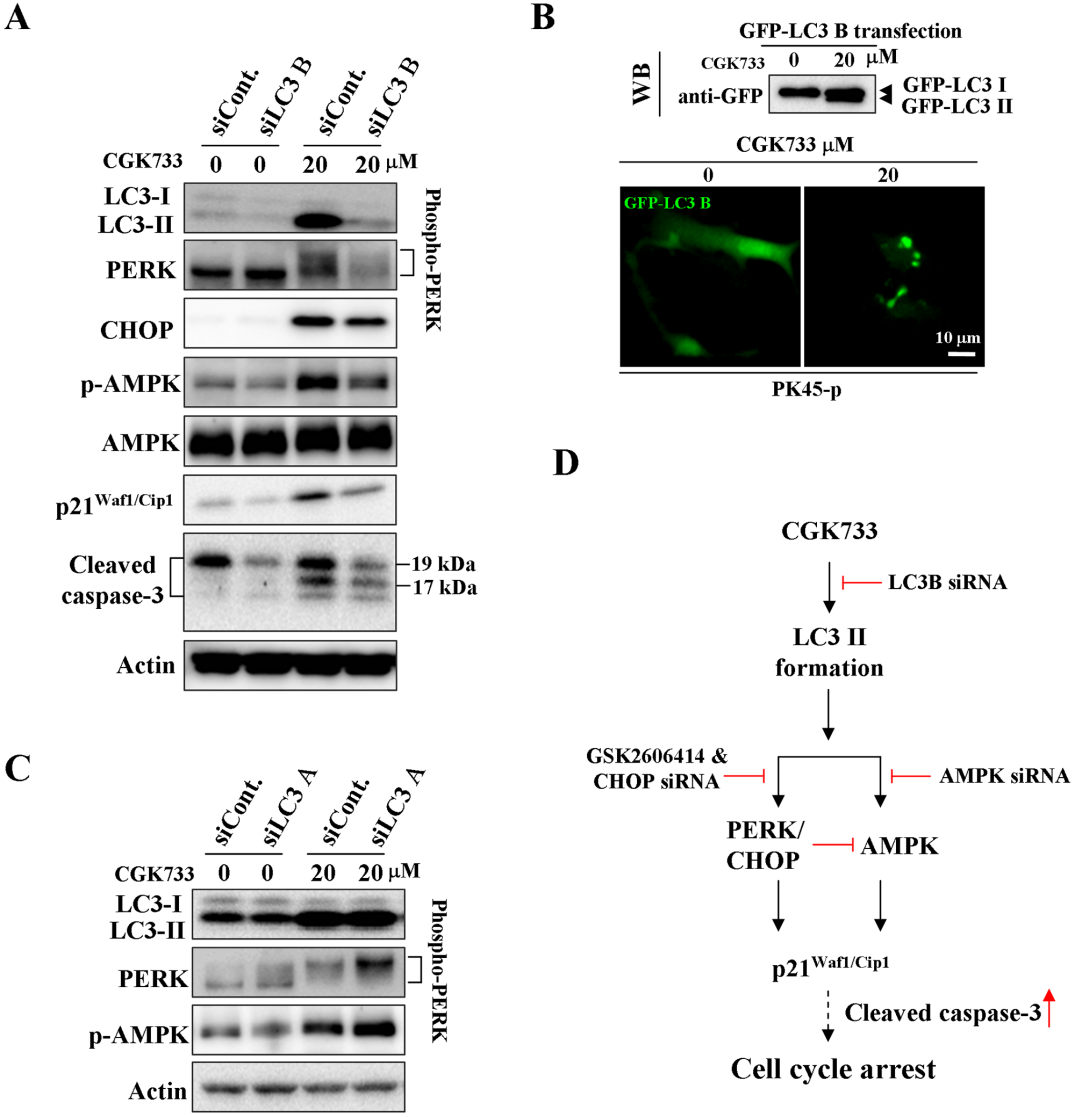
CGK733 induced LC3 II formation and the activation of AMPK and PERK/CHOP through LC3 B **A.** PK45-*p* cells were treated with CGK733 for 6 h after transfection with LC3 B siRNA for 48 h. **B.** PK45-*p* cells were treated with CGK733 for 6 h after transfection with EGFP-LC3 B plasmid for 48 h. **C.** PK45-*p* cells were treated with CGK733 for 6 h after the transfection with LC3 A siRNA for 48 h. **D.** The scheme indicates that CGK733 induced the expression of p21^Waf1/Cip1^ through the LC3 B-mediated AMPK and PERK/CHOP activation.

We next tested whether CGK733-induced LC3 II formation is required for the activation of AMPK and PERK/CHOP. Knockdown of LC3 B (but not LC3 A) significantly inhibited the CGK733-induced LC3 II and GFP-LC3 puncta formation (Figure 4A and 4B). The activation of AMPK and PERK/CHOP, as well as p21^Waf1/Cip1^ expression following treatment with CGK733 were simultaneously blocked by LC3 B knockdown Figure 4C. However, The CGK733-induced AMPK and PERK/CHOP activation was further enhanced after knockdown of LC3 A. This indicated that LC3 A may have the opposite role of LC3 B in the modulation of the AMPK and PERK/CHOP signaling pathways. These results reveal that CGK733-triggered LC3 II formation is LC3 B-dependent and it is an initial event upstream of both AMPK and PERK/CHOP signaling pathways.

## DISCUSSION

In the present study the treatment of pancreatic cancer cell lines with CGK733 for 6 h resulted in the expression of LC3-II and formation of LC3-II puncta in a dose-dependent manner. However, these CGK733-induced LC3-puncta appear to be exclusive of canonical autophagy as they were accompanied by unaltered expression of p62 and total protein ubiquitination, and presented reduced p62-LC3 colocalization or binding. The independence of p62 (and its related protein NBR1) from LC3 was previously shown in endoplasmic reticulum (ER)-associated autophagosome formation [19].

Moreover, CGK733 treatment increased expression of phospho-AMPK (at Thr 172), PERK, p21^Waf1/Cip1^ and cleaved caspase-3. Neither AMPK knockdown nor PERK inhibition or CHOP knockdown could alter the CGK733-induced LC3 II formation. However, these inhibitions blocked the CGK733-induced expression of p21^Waf1/Cip1^ and caspase-3 cleavage. This indicates that while these two events are a direct consequence of CGK733 on the AMPK and PERK/CHOP signaling pathways, the observed LC3 II formation is not.

The activation of p21^WAF1/CIP1^ by CGK733 is the Gemcitabine-resistant pancreatic cancer cell lines tested is very interesting as p21^WAF1/CIP1^ is known to be involved in mediating p53-dependent cell cycle arrest following DNA damage [20–22] but can also inhibit apoptosis [23–27]. This means that while its former function makes it a tumor suppressor, its latter function makes it an oncogene. It has been shown that p21^WAF1/CIP1^ acts as a tumor suppressor in the colon, liver and renal cancers of mice [28, 29] while being an oncogene in lymphomas, gliomas, rectal and esophageal squamous cell carcinomas [23, 30–36]. Induction of p21^WAF1/CIP1^ by chemotherapy agents (Irinotecan or doxorubicin) was shown to lead to senescence in mesothelial, breast, lung and colon carcinomas, thus blocking apoptosis [37]. Of particular interest is that when senescent breast, lung, and colon carcinoma cells were treated with CGK733, it led to suppression of p21^WAF1/CIP1^ [12]. However, PK59 cells are not senescent and CGK733 induces them to shift to S phase (Supplementary Figure S3). This means that p21^WAF1/CIP1^ is acting as an oncogene.

In the present study it was possible to show that CGK733-induced LC3 II and GFP-LC3 puncta formation are due to LC3 B but not LC3 A, and while LC3 B knockdown blocked the CGK733-activation of AMPK and PERK/CHOP as well as expression of p21^Waf1/Cip1^, these were in contrast enhanced after LC3 A knockdown. Thus, the major original result from this research is that in the Gemcitabine-resistant pancreatic cancer cell lines tested, the LC3 B-dependent GK733-induced LC3 II formation is an initial event upstream of both AMPK and PERK/CHOP signaling pathways. It is hypothesised that this is possibly via binding of LC3 with ULK1 [38, 39], which brings about the binding with AMPK and activation of PERK.

The role of AMPK in mediating autophagy and inducing LC3 B has been previously reported, either via FoxO3a transcription factor activation or direct inhibition of AMPK by a chemical inhibitor or siRNA [40, 41]. Similarly, the link between LC3 B and the PERK/CHOP activity has been previously shown with knockdown of CHOP inhibiting LC3 B-II expression in Hepatitis C virus infected cells [42]. Another study reported a reduction in the number of GFP-LC3 B dots in GFP-LC3 B-transfected HT29 cells treated with the Ca^2+^ ion pump protein inhibitor Thapsigargin [43]. However this is the first report of LC3 B being upstream of both AMPK and PERK/CHOP signaling pathways (Figure 4D).

## MATERIALS AND METHODS

### Cell culture

The Gemcitabine-resistant pancreatic cancer cell lines (PK54-p and PK59) were cultured in Roswell Park Memorial Institute 1640 medium, RPMI 1640 (GIBCO, 05918), supplemented with 10% heat-inactivated fetal bovine serum (FBS; GIBCO, 26140-079), and 2 mM L-glutamine and incubated at 37°C in a humidified incubator containing 5% CO_2_.

### Materials

CGK733 (sc-202964), 3-MA (sc-205596) and Compound C (sc-200689) were purchased from Santa Cruz Biotechnology. Chloroquine (C6628) and rapamycin (R8781) were purchased from Sigma-Aldrich. GSK2606414 (G797800) was purchased from Tront Research Chemicals Inc. The AMPK (sc-45312), LC3A (sc-106197), LC3B (sc-43390), CHOP (sc-35437) and control (sc-37007) siRNAs were purchased from Santa Cruz Biotechnology. The p-AMPK (4184S), LC3 A/B (4108S, WB/IP), cleaved caspase-3 (9661) and CHOP (2895S) antibodies were purchased from Cell Signaling Technology. The LC3 A/B (ab58610, IF) and PERK (ab65142) antibodies were purchased from Abcam. The AMPK (07–350) antibody was purchased from Millipore (Bedford, MA). The P62/SQSTM1 (P0067) antibody were purchased from Sigma-Aldrich. The Actin (sc-1616) and p21^Waf1/Cip1^ (sc-65595) antibodies were purchased from Santa Cruz Biotechnology.

### Western blot

The cells were suspended in lysis buffer on ice for 1 h [44]. Equal amounts of protein (20 μg) were resolved by 5–20% SDS-polyacrylamide gel (Wako, SuperSep Ace 194–15021) and then transferred onto PVDF membrane (Immobilon, IPVH-00010). The membrane was incubated with the appropriate primary antibody at 4°C overnight and a horseradish peroxidase (HRP)-conjugated secondary antibody for 1 h at room temperature [45]. The immunoblots were visualized with a chemiluminescence reagent (Wako, Immunostar 290–69904).

### Immunofluorescence

Cells were cultured on coverslips in 12 well plates at a density of 1 × 10^5^ cells per well. Cells were fixed using fresh 3.7% paraformaldehyde in phosphate buffered saline (PBS) for 30 min and permeabilized with 0.1% Triton X-100 for 15 min. After washing with PBS they were incubated in blocking solution (1% goat serum or 1% donkey serum in PBS with 0.1% Tween 20) for 1 h at room temperature. Cells were treated with anti-LC3 A/B primary antibody (ab58610) in blocking solution overnight at 4°C and with a secondary antibody for 1 h at room temperature. Cell nuclei were counter-stained with 1.43 μM of DAPI for 5 minutes. Confocal images were obtained using Nikon Plan Apo 60X/1.40 objective, BZ-9000 series (BIOREVO) and BZ-II Viewer software (Keyence, Osaka, Japan) by an operator who was unaware of the experimental condition.

### Transient transfection

Cells were seeded and incubate at 37° C in a CO_2_ incubator until the cells were 70% confluent. Cells were transfected with validated siRNA, TagGFP2-LC3 Lentivirus (Millipore, 17–10193) or EGFP-LC3 B (Addgene, 11546), following the manufacturer's siRNA Transfection Protocol (Santa Cruz Biotechnology), Lentiviral Transduction Protocol (Millipore) or Xfect™ Transfection Reagent Protocol (Clontech Laboratories, PT5003-2), respectively.

### Immunoprecipitation

A dynabeads-antibody complex was prepared using an anti-LC3 A/B primary antibody (4108S) and Immunoprecipitation Kit Dynabeads Protein G (Novex, 10007D) by following the manufacturer`s Immunoprecipitation Protocol (Novex by Life Technologies). LC3 and p62 were detected by Western blotting with anti-LC3 A/B (4108S) and anti-p62/SQSTM1 (Sigma-Aldrich, P0067) primary antibodies.

### Cell Sorting

Cells were cultured in 10 cm dishes and treated with 20 μM CGK733 for 6 h. After trypsinization, the cell pellet was fixed in 70% ethanol for 2 hours at 4°C. The cells were then incubated in 0.25 mg/ml RNase/PBS at 37°C for 1 hour and stained with propidium iodide (PI; 50 μg/ml) for 30 min at 4°C. The stained cells were analyzed by the Cell Lab Quanta SC (Beckman coulter, Inc., Brea, CA). The electronic volume was used for gating so as to reduce background noise. Up to 10,000 cells were counted and the PI fluorescence was detected by the FL-3 gate.

## SUPPLEMENTARY FIGURES


