# Supplementary material for: CGK733-induced LC3 II formation is positively associated with the expression of cyclin-dependent kinase inhibitor p21Waf1/Cip1 through modulation of the AMPK and PERK/CHOP signaling pathways
Source: Oncotarget. 2015 Oct 13;6(37):39692–701. doi: 10.18632/oncotarget.5625 (PMC4741855; doi:10.18632/oncotarget.5625)
Supplement: Supplementary file 1 [file oncotarget-06-39692-s001.pdf]

SUPPLEMENTARY FIGURES

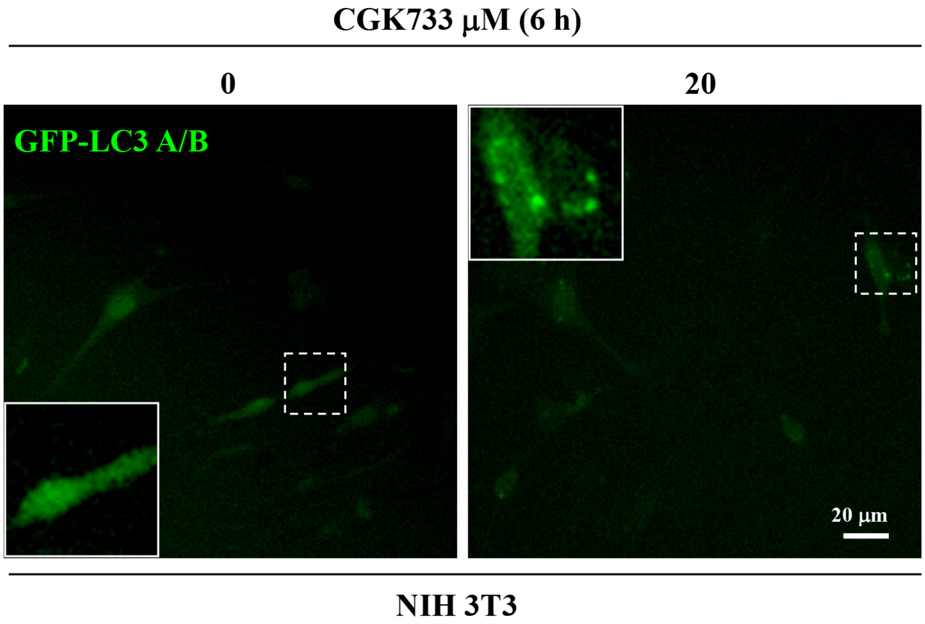

Supplementary Figure S1: CGK733 induces LC3-puncta formation in embryonic fibroblast. Embryonic fibroblast NIH 3T3 cells were transfected with TagGFP2-LC3 Lentivirus for 48 h and then treated with CGK733 for 6 h before observation by fluorescent microscopy.

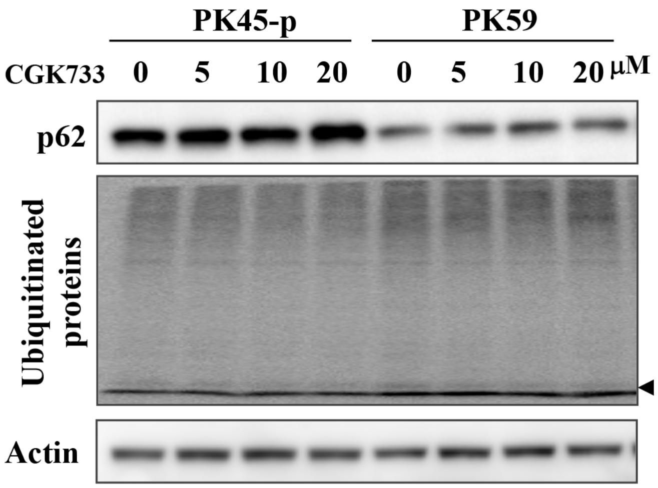

Supplementary Figure S2: CGK733 did not trigger p62-mediated degradation. PK45-p and PK59 cells were treated with CGK733 for 6 h before being processed western blot analysis using antibodies against p62, ubiqutin and actin.

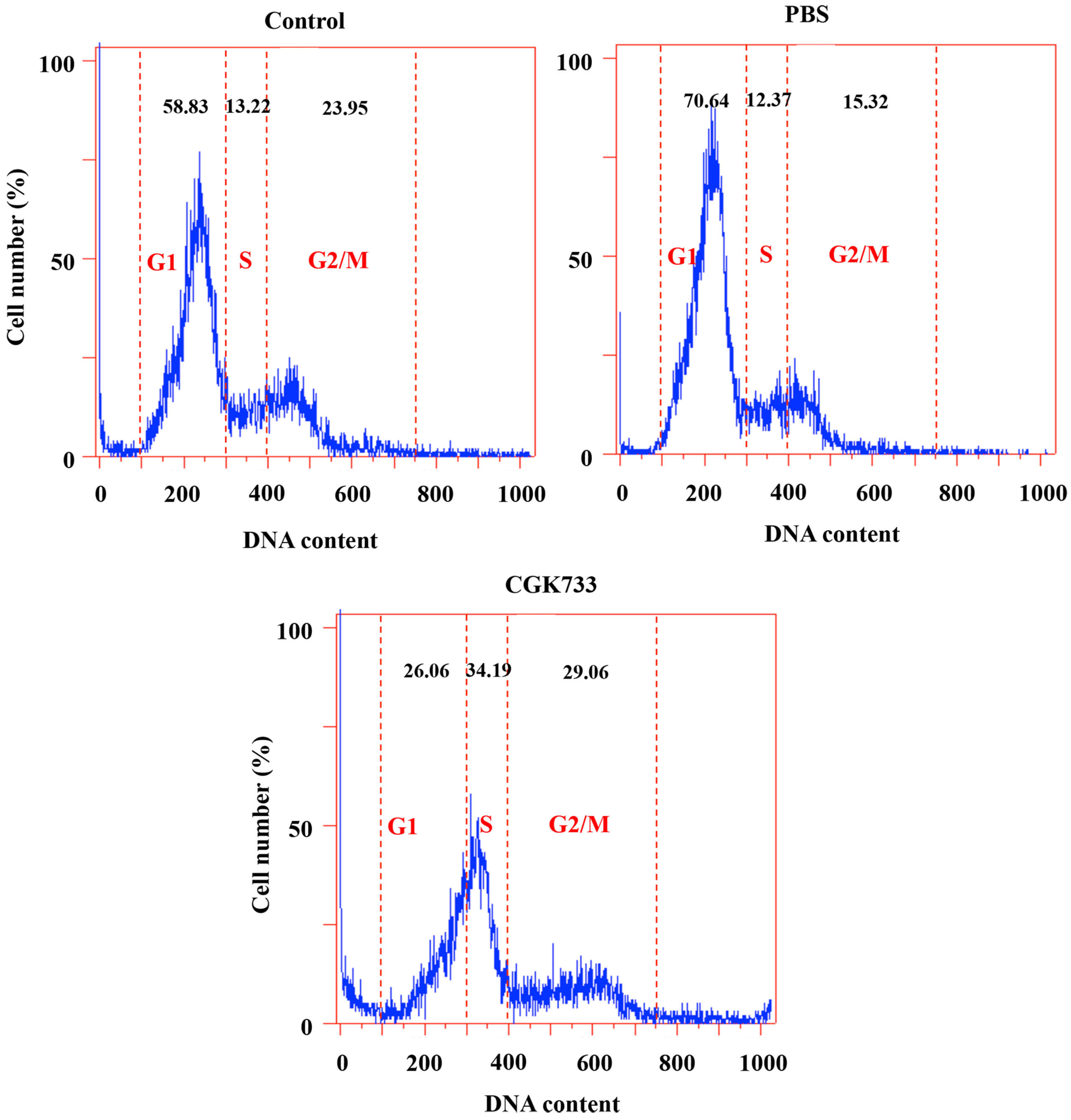

Supplementary Figure S3: CGK733 induces cell cycle arrest at S phase. PK59 cells were treated with CGK733 for 6 h before analysis by Flow Cytometry.
